# Corps étranger intra vésical: un cas exceptionnel

**DOI:** 10.11604/pamj.2016.23.202.4564

**Published:** 2016-04-20

**Authors:** Younes Essatara, Hicham Benazzouz

**Affiliations:** 1 Clinique Urologique A, CHU IBN Sina, Rabat, Maroc

**Keywords:** Corps étranger, jeune fille, crayon, Foreign body, girl, pencil

## Image en médecine

De nombreux articles de la littérature médicale rapportent des cas d'introduction de corps étranger dans la filière urogénitale. La plupart des cas sont associés à des désordres psychiatriques, à des cas de toxicomanie, ou dans un but de stimulation sexuelle. La vessie semble être un site inaccessible pour l'introduction de corps étranger surtout chez l'homme, cependant tous les objets concevables ont été insérés dans la vessie à travers l'urètre et chacun a posé des problèmes diagnostiques et thérapeutiques particuliers. Une jeune fille de 19 ans, s'est présentée aux urgences affirmant qu'elle s'est introduite il y'a 7 jours, dans un but érotique un crayon de couleur au niveau du méat. La patiente consciente, lucide, rapportait une notion d'hématurie et de douleur hypogastrique. L'examen clinique ne retrouvait pas le crayon au niveau du méat et ne montrait ni hématurie, ni contracture abdominale. Dans un but d'orientation diagnostique un arbre urinaire sans préparation a objectivé une opacité linéaire rappelant la forme d'un crayon se projetant sur l'aire vésicale, l’échographie vésicale après remplissage vésical a retrouvé une image hyperéchogèneintravésicale confirmé par TDM mettant en évidence le crayon avec aspect longitudinale hyperdense effilé à son extrémité. Une cystoscopie pratiquée dans un but diagnostique et thérapeutique a permis de voir le crayon, de témoigner aussi de l'intégrité urétrale et vésicale et de retirer le corps étranger. La patiente fut adressée en consultation de psychiatrie pour avis et complément de prise en charge.

**Figure 1:**
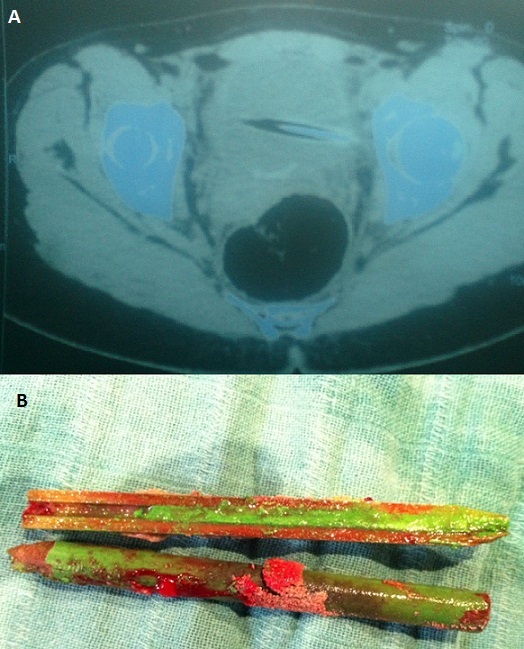
A) aspect TDM du corps étranger intrvesical, hyperdense, éffilé à son extrémité; B) aspect du corps (crayon) après extraction par cytoscopie

